# Supplementary material for: Angiotensin II receptor blockers and oral squamous cell carcinoma survival: A propensity-score-matched cohort study
Source: PLoS One. 2021 Dec 2;16(12):e0260772. doi: 10.1371/journal.pone.0260772 (PMC8638984; doi:10.1371/journal.pone.0260772)
Supplement: S1 Table — (DOCX) [file pone.0260772.s001.docx]

S1 Table. Baseline characteristics of OSCC patients before and after propensity-score matching.

| **Characteristics** | **Before Propensity Score Matching** | | | **After Propensity Score Matching** | | | |  |
| --- | --- | --- | --- | --- | --- | --- | --- | --- |
|  | None-users  (Control)  n=5311 | ARB-users ≥ 180 days  (Intervention)  n=362 | Standardized  Mean  Difference | | None-users  (Control)  n=357 | ARB-users ≥ 180 days  (Intervention)  n=357 | Standardized  Mean  Difference | |
| **Propensity Score**  **Gender** | 0.025(0.016-0.043) | 0.158(0.054-0.426) | 1.1915 | | 0.156(0.054-0.407) | 0.157(0.054-0.419) | 0.0079 | |
| Female | 450(8.5%) | 27(7.5%) | 0.0374 | | 28(7.8%) | 27(7.6%) | 0.0105 | |
| Male | 4861(91.5%) | 335(92.5%) | 0.0374 | | 329(92.2%) | 330(92.4%) | 0.0105 | |
| **Age (IQR)**  **Pathological**  **AJCC 7^th^ staging** | 52(45-59) | 58(52-66) | 0.6026 | | 59(52-66) | 58(51-66) | 0.0729 | |
| I | 1433(27.0%) | 122(33.7%) | 0.1464 | | 112(31.4%) | 119(33.3%) | 0.0419 | |
| II | 1212(22.8%) | 106(29.3%) | 0.1475 | | 108(30.3%) | 104(27.1%) | 0.0245 | |
| III | 720(13.6%) | 45(12.4%) | 0.0335 | | 49(13.7%) | 45(12.6%) | 0.0331 | |
| IVa & IVb | 1946(36.6%) | 89(24.6%) | 0.2637 | | 88(24.7%) | 89(24.9%) | 0.0065 | |
| **Comorbidities**  Diabetes mellitus  Hyperlipidemia | 656(12.4%)  404(7.6%) | 201(55.5%)  188(51.9%) | 1.0234  1.1071 | | 203(56.9%)  171(47.9%) | 196(54.9%)  183(51.2%) | 0.0394  0.0672 | |
| **Diagnostic Year**  **of OSCC**  2007 | 502(9.5%) | 47(13.0%) | 0.112 | | 51(14.3%) | 46(12.9%) | 0.0408 | |
| 2008 | 495(9.3%) | 30(8.3%) | 0.0364 | | 33(9.2%) | 30(8.4%) | 0.0296 | |
| 2009 | 519(9.8%) | 38(10.5%) | 0.024 | | 37(10.4%) | 38(10.6%) | 0.0091 | |
| 2010 | 499(9.4%) | 33(9.1%) | 0.0096 | | 31(8.7%) | 33(9.2%) | 0.0196 | |
| 2011 | 527(9.9%) | 38(10.5%) | 0.019 | | 31(8.7%) | 38(10.6%) | 0.0663 | |
| 2012 | 535(10.1%) | 42(11.6%) | 0.0492 | | 43(12.0%) | 42(11.8%) | 0.0086 | |
| 2013 | 470(8.9%) | 33(9.1%) | 0.0093 | | 34(9.5%) | 32(9.0%) | 0.0193 | |
| 2014 | 474(8.9%) | 32(8.8%) | 0.003 | | 24(6.7%) | 31(8.7%) | 0.0735 | |
| 2015 | 511(9.6%) | 31(8.6%) | 0.0368 | | 31(8.7%) | 29(8.1%) | 0.0202 | |
| 2016 | 523(9.9%) | 27(7.5%) | 0.085 | | 26(7.3%) | 27(7.6%) | 0.0107 | |
| 2017 | 256(4.8%) | 11(3.0%) | 0.0917 | | 16(4.5%) | 11(3.1%) | 0.0734 | |

Abbreviations: AJCC, American Joint Committee on Cancer; ARB, angiotensin II receptor blocker; OSCC, oral squamous cell carcinoma; IQR, interquartile range
