# Supplementary material for: Angiotensin II receptor blockers and oral squamous cell carcinoma survival: A propensity-score-matched cohort study
Source: PLoS One. 2021 Dec 2;16(12):e0260772. doi: 10.1371/journal.pone.0260772 (PMC8638984; doi:10.1371/journal.pone.0260772)
Supplement: S2 Table — (DOCX) [file pone.0260772.s002.docx]

S2 Table. The included angiotensin II receptor blockers and numbers in our study

| **Medication** | **Number of**  **included patients** |
| --- | --- |
| Irbesartan 150mg | 8 |
| Irbesartan 150mg+HCTZ 12.5 mg | 4 |
| Irbesartan 300mg | 42 |
| Irbesartan 300mg+HCTZ 12.5 mg | 11 |
| ------------------------------------------------------------------------------------------------------ | |
| Losartan potassium 50mg | 67 |
| Losartan 50mg+HCTZ 12.5mg | 1 |
| Losartan potassium 100mg | 2 |
| Losartan 100mg+HCTZ 12.5 mg | 11 |
| ------------------------------------------------------------------------------------------------------ | |
| Olmesartan 20mg | 12 |
| Amlodipine 5mg+Olmesartan medoxomil 20mg | 10 |
| Amlodipine 5mg+Olmesartan medoxomil 40mg | 21 |
| Amlodipine 5mg+Olmesartan 20mg+HCTZ 12.5mg | 7 |
| ------------------------------------------------------------------------------------------------------ | |
| Valsartan 80mg | 1 |
| Valsartan 160mg | 84 |
| Valsartan 160mg+HCTZ 12.5 mg | 15 |
| ------------------------------------------------------------------------------------------------------ | |
| Azilsartan medoxomil 40mg | 7 |
| ------------------------------------------------------------------------------------------------------ | |
| Candesartan 8mg | 41 |
| Candesartan 16mg+HCTZ 12.5 mg | 4 |
| ------------------------------------------------------------------------------------------------------ | |
| Telmisartan 40mg | 7 |
| Telmisartan 80mg | 2 |

Abbreviations: HCTZ, hydrochlorothiazide
