# Supplementary material for: Angiotensin II receptor blockers and oral squamous cell carcinoma survival: A propensity-score-matched cohort study
Source: PLoS One. 2021 Dec 2;16(12):e0260772. doi: 10.1371/journal.pone.0260772 (PMC8638984; doi:10.1371/journal.pone.0260772)
Supplement: Attachment — Submitted filename: 20210927_Rebutal-letter opinions.docx [file pone.0260772.s003.docx]

Dear editor, reviewers, and journal office:

Thank you for reviewing our manuscript entitled “Angiotensin II receptor blockers and oral squamous cell carcinoma survival: A propensity-score-matched cohort study.” The editorial formatting comments and the suggestions of the reviewer are greatly appreciated.

The funding-related text from the manuscript has been removed, and our Funding Statement remained consistently without modification. Besides, I applogized that I had possibliy chosen a worng statement about the Data Availability. The data cannot be shared publicly because it is owned by Chang Gung Medical Branches and authors do not have permission to share the data. Data are available from the Department of Medical Research and Development for researchers who meet the criteria for access to confidential data.

This revised version has been modified according to the comments given by your panel of reviewers and editor. In order to reply to the specific comments of the reviewers, we have revised the manuscript. Below please find our point-by-point responses to each comment made by the reviewers.

Lastly, we would like to thank you once again for providing us the opportunity to improve our manuscript. We hope that these revisions are acceptable and that the manuscript is now suitable for publication in the PLOS ONE.

Sincerely

Tai-Jan Chiu, MD

Department of Hematology-Oncology, Kaohsiung Chang Gung Memorial Hospital and Chang Gung University College of Medicine, Kaohsiung, Taiwan.

123 Ta-Pei Road, Niao-Song District, Kaohsiung, 833, Taiwan

E-mail: kuerten@cgmh.org.tw, chiutaijan@gmail.com

**RE: “Angiotensin II receptor blockers and oral squamous cell carcinoma survival: A propensity-score-matched cohort study.”**

We would like to thank the reviewers for the thorough reading of our manuscript as well as their valuable comments. We have followed their comments closely and feel that their suggestions have further strengthened the manuscript. Below are our point-by-point responses.

**Response to Reviewer’s Comments:**

*Review 1:*

The authors reported that ARB use for more than 180 days is associated with an increased survival rate and is a positive, independent prognostic factor in patients with oral squamous cell carcinoma (OSCC) in their propensity-score-matched cohort from the registry database. It is interesting but also arguable results. As the authors commented and listed other studies reporting the association between the ARBs and cancers (Table 7), it is still unclear whether the ARBs have a survival benefit in most cancers. For now, it is evident that treatment with an ARB medication does not increase the risk of cancer (FDA report <https://www.fda.gov/drugs/drug-safety-and-availability/fda-drug-safety-communication-no-increase-risk-cancer-certain-blood-pressure-drugs-angiotensin>). Even though they found through the skilled analysis from their propensity-score-matched cohort, their findings are the association between ARBs use and survival in OSCC patients NOT the causal relationship. So they may not conclude “ARBs have survival benefit in OSCC patients”. It should be very prudent to this conclusion. In this regard, I recommend to erase the final parenthesis “These findings highlight the clinical usefulness of ARBs in OSCC patients with advanced disease” in conclusion. And the authors had better to add some comments on this critical issue regarding the causal relationship.

**Response:** Dear reviewer, I agree with your precise comment. I deleted the final parenthesis according to your opinion and changed it to “A further two-arm study should be conducted to confirm the clinical usefulness of ARBs in OSCC patients.” **(Page 2 Abstract; Page 22 Conclusion).** We also added some comments on this issue in the Discussion section as the following: “However, there was no consistent conclusion on whether ARBs have a survival benefit in patients coexisting with malignancies. One of the main reasons was the discrepancy in confounding control across these studies. Unknown confounding and selection bias might exist in these retrospective studies with different study design, indicating the findings are only the association between ARBs use and survival. Future two-arm controlled study should be conducted for strengthening the causal relationship.” **(Page 17)**

*Review 2:*

1. There is no data of blood pressure. Whether the patients take ARB or not, whether the patients have hypertension or not, whether the patients have cancer or not, hypertension is the very important factor of mortality. Therefore, the data of blood pressure of two groups is very important and if authors prove the usefulness of ARB in the context of increasing survival rate, the authors must show the data of blood pressure and when the authors analyze the data, the authors must correct the data of blood pressure.

**Response:** Dear reviewer, I agree with your opinion very well, and I think what you are concerned about is very important. However, I am sorry that we didn’t have the complete blood pressure (BP) data for each person in our database. The most representative BP comes from the daily measurement by the patient at home, or the BP measured in the clinic at least. However, we only had the BP measured during the hospitalization, which was available for 616 patients. The most crucial limitation from the hospitalization BP was the incomplete data for all patients and many confounding factors existing in this data. For example, patients staying in the hospital might suffer from acute disease, which induced unstable BP measurement. Some patients might even be in a critical status, which caused a lower BP result. Because of the reasons above, I deem it as a major limitation in our study, and put it into our discussion. I hope you could accept my explanation.

We agree your comment is crucial for future study. Therefore, I analyzed the hospitalization BP regardless of the reasons we mentioned above. The data units of systemic blood pressure (SBP) level below 80 mmHg were deleted first, which I deemed probable data of critical status. Then I collected and averaged all the SBP data for each person (n=616). Afterward, the SBP data were classified into normal and abnormal by BP=140 mmHg. We put the variate into our Cox regression model and found no statistical effect on overall survival (HR _high BP vs. normal BP_ = 0.79, 95% CI = 0.54-1.14). A similar result was found if we changed the divide by BP=120 mmHg (HR _high BP vs. normal BP_ = 0.73, 95% CI = 0.48-1.11). However, we can’t jump to the conclusion that BP was not associated with survival because the above analysis was based on an incomplete and unrepresentative hospitalization BP database.

In conclusion, a complete representative BP measurement should be taken into account in a future study. Therefore, I summarized the above explanation and added it into the discussion section: “Lastly, blood pressure, a crucial variate in an anti-hypertensive medication study, was not taken into account in our research. The main reasons were that complete data of blood pressure was not available in our database, and many confounding factors existed in the residual database as well. However, taking representative blood pressure data into analysis should be considered in future studies.” **(Page 21)**

2. The authors reveal that all patients using ARB have hypertension, but only 37.5 % of the patients not using ARB have hypertension. If the authors discuss the usefulness of ARB in the context of increasing survival rate of OSCC patients, the authors need to demonstrate whether the reason of the usefulness of ARB is class effect or antihypertensive effect.

**Response:** Dear reviewer, your comment about survival benefit might be from ARB use probably from the antihypertensive effect instead of the class effect. It is a question not easy to answer. Here, I assumed that the survival of patients with ARB use would not be superior to those without hypertension if the antihypertensive effect plays a significant role in survival. This assumption is based on the previous research (Ref 1), in which patients with hypertension had poor long-term survival than patients without hypertension. In other words, the class effect of ARBs on the survival benefit in oral cancer patients could be approved if the survival of patients with ARB use (hypertension group) is superior to those without hypertension.

We tested this hypothesis and calculated the crude and adjusted hazard ratio, including a 95% confidence interval for overall survival in patients with ARBs use (n=357) relative to patients without hypertension (n=223). Notably, 180-day ARB use was consistently associated with improved OS with or without adjustment. (Crude HR_ARB users vs. non-HTN_ = 0.70, 95% CI = 0.50-0.99; adjusted HR_ARB users vs. non-HTN_ = 0.67, 95% CI = 0.48-0.95). The above findings displayed the possible anti-cancer effect of ARBs in oral cancer patients. In addition, there is no evidence that other antihypertensive agents suppress cancer development or progression, which had been described in the Limitation section. The class effect of ARBs was therefore indirectly approved.

We appreciate your incisive comments. The above statements were concisely added into our Discussion section as the following:” Some might argue that the survival benefit from ARB use was probably from the antihypertensive effect instead of the anti-cancer effects. According to a previous study, patients with hypertension had poor long-term survival than those without hypertension [28]. However, in our study, 180-day ARB use in patients with hypertension was consistently associated with improved OS compared to patients without hypertension. (Crude HR_ARB users vs. non-HTN_ = 0.70, 95% CI = 0.50-0.99; adjusted HR_ARB users vs. non-HTN_ = 0.67, 95% CI = 0.48-0.95). The above findings displayed the possible anti-cancer effect of ARBs in oral cancer patients.” **(Page 19)**

Ref 1. Andersson OK, Almgren T, Persson B, Samuelsson O, Hedner T, Wilhelmsen L. Survival in treated hypertension: follow up study after two decades. BMJ. 1998;317(7152):167-171. doi:10.1136/bmj.317.7152.167

3. There are various pathological types of oral cancer. Why do the authors choose only squamous cell carcinoma? Why do the authors exclude non-squamous cell carcinoma during the first stage? I can understand that the authors analyze by type of pathological findings. However, I cannot understand excluding the patient of non-squamous cell carcinoma. I think the authors must also analyze for the patients of non-squamous cell carcinoma.

**Response:** Dear reviewer, what you had concerned about is reasonable. However, according to the past epidemiology study of oral cancer in Asia, the majority (84-97%) of oral cancer is squamous cell carcinoma (Ref 1). One native study in Taiwan also showed that squamous cell carcinoma type occupied 93.1% of oral cancer (Ref 2). Because of the epidemiologic characteristics in Taiwan, we focused on the major squamous cell carcinoma type to prevent accidental confounding from other types. I hope you can accept this explanation.

Ref 1. Krishna Rao SV, Mejia G, Roberts-Thomson K, Logan R. Epidemiology of oral cancer in Asia in the past decade--an update (2000-2012). Asian Pac J Cancer Prev. 2013;14(10):5567-77. doi: 10.7314/apjcp.2013.14.10.5567.

Ref 2. Huang CC, Ou CY, Lee WT, Hsiao JR, Tsai ST, Wang JD. Life expectancy and expected years of life lost to oral cancer in Taiwan: a nation-wide analysis of 22,024 cases followed for 10 years. Oral Oncol. 2015 Apr;51(4):349-54. doi: 10.1016/j.oraloncology.2015.01.001. Epub 2015 Jan 17.

4. Can’t the cancer duration at the start of medication of ARB or antihypertensive agents be a confounding factor?

**Response:** Yes, we agree with your opinion. The duration between initial cancer diagnosis and the start point of ARB use could be a confounding factor, which was often considered an immortal time bias. To prevent such a bias, we had considered “the diagnostic year of oral cancer” as a covariate in both groups when performing propensity score matching. We had mentioned this part in the “Statistical Analyses” section as the following:” Propensity scores were calculated using a logistic regression model with sex, age, pathological AJCC stage, comorbidities, and the diagnostic year of OSCC as covariates (S1 Table). … Because the diagnostic years were matched in both groups, the calculated survival time in non-users started from the same day as its match to make the comparison between the two groups fair.” **(Page 6 Statistical Analyses)**

5. Why do the authors decide the cut off period of receiving ARB to more than 180 days or not?

**Response:** Thank you for the excellent comment. Why we decided the cut-off period of 180 days was based on the previous research. The main reason is to control for protopathic bias, and therefore some studies have incorporated the concept of lag-time into their exposure definition. The best lag-time to be applied was also studied, which around six months were the most appropriate period for the assessment of drug exposure (Ref 1). We didn’t explain it clearly in the original manuscript, and therefore, we now added the supplement as the followings: “In addition, the best lag-time to be applied in studies accordingly was around six months, which was the most appropriate period for the assessment of drug exposure [29].” **(Page 20)**

There was another direct and practical reason. We had run a pre-analysis Kaplan-Meier Method, which considers the possible effects of at least three months (90days) or six months (180 days). The figures are shown below, and the preliminary result showed that only receiving ARBs for more than 180 days had a statistical survival benefit.

Ref 1. Tamim H, Monfared AA, LeLorier J. Application of lag-time into exposure definitions to control protopathic bias. Pharmacoepidemiol Drug Saf. 2007 Mar;16(3):250-8. doi: 10.1002/pds.1360. PMID: 17245804.
