# Supplementary material for: Angiotensin II receptor blockers and oral squamous cell carcinoma survival: A propensity-score-matched cohort study
Source: PLoS One. 2021 Dec 2;16(12):e0260772. doi: 10.1371/journal.pone.0260772 (PMC8638984; doi:10.1371/journal.pone.0260772)
Supplement: Attachment — Submitted filename: 20211104_Rebutal-letter opinions.docx [file pone.0260772.s004.docx]

Dear editor, reviewers, and journal office:

Thank you for reviewing our manuscript entitled “Angiotensin II receptor blockers and oral squamous cell carcinoma survival: A propensity-score-matched cohort study.” The comments of the reviewers are greatly appreciated.

This revised version has been modified according to the comments given by your panel of reviewers and editor. In order to reply to the specific comments of the reviewers, we have revised the manuscript, added essential references (Ref. 27, 28, 29), and deleted some unnecessity (previous Ref. 26, 31). Below please find our point-by-point responses to each comment made by the reviewers.

Lastly, we would like to thank you once again for providing us the opportunity to improve our manuscript. We hope that these revisions are acceptable and that the manuscript is now suitable for publication in the PLOS ONE.

Sincerely

Tai-Jan Chiu, MD

Department of Hematology-Oncology, Kaohsiung Chang Gung Memorial Hospital and Chang Gung University College of Medicine, Kaohsiung, Taiwan.

123 Ta-Pei Road, Niao-Song District, Kaohsiung, 833, Taiwan

E-mail: kuerten@cgmh.org.tw, chiutaijan@gmail.com

**RE: “Angiotensin II receptor blockers and oral squamous cell carcinoma survival: A propensity-score-matched cohort study.”**

We would like to thank the reviewers for the thorough reading of our manuscript as well as their valuable comments. We have followed their comments closely and feel that their suggestions have further strengthened the manuscript. Below are our point-by-point responses.

**Response to Reviewer’s Comments:**

*Review 1:*

The authors addressed adequately my comments. I think the paper revised properly with adequate answers to reviewer's comments.

**Response:** Dear reviewer, we thanked again for your thorough reading of our manuscript as well as the valuable comments.

*Review 2:*

Most of your response of the authors can be understanded, however, some responses seem to be arguable. About my question No.1-2, your responses are very well-formed, and your analysis about 616 patients of available information of BP measurements is very important data, however it seems that the reason of survival benefit of using ARB in oral cancer patients is not always anti-cancer effect of ARBs. Even if the effect of survival benefit of using ARB is not dependent on blood pressure, the reasons of survival benefit of using ARB are thought of as several organ protective effect, such as cardioprotective effect or renal protective effect. Therefore, the author should delete enrollment of possible anti-cancer effect of ARBs, and should describe another consideration about survival benefit of using ARB in oral cancer patients.

**Response:** Dear reviewer, I agree with your opinion addressed” Even if the effect of survival benefit of using ARB is not dependent on blood pressure, the reasons of survival benefit of using ARB are thought of as several organ protective effect, such as cardioprotective effect or renal protective effect.” I think what you mentioned explained the main findings in our result, which the survival benefit only showed statistically significant in OS, but not in DSS. Therefore, some discussion and reference about this issue were added accordingly.

However, directly deleting possible anti-cancer effect of ARBs concerns me. In our discussion, the evidence of anti-cancer mechanism did exist in the molecular level in head and neck cancer (Ref 1,2). In addition, a newly published article in *Cancer* (Ref 3) also proved that ARB use provided clinical survival benefits (both OS and DSS) with specific anti-tumor molecular signaling in nasopharyngeal carcinoma, which indicated that ARB might have anti-cancer effects on HNSCC. In our article, the anti-cancer effect didn’t reach a statistically significance (DSS, HR_ARB users vs. non-users_ = 0.73, 95% CI = 0.48–1.10), but the effect size was nevertheless similar to OS (HR_ARB users vs. non-users_ = 0.73, 95% CI = 0.53-0.99). I would rather interpret that there might probably exist an anti-cancer effect, yet with more variability, which the conclusion of anti-cancer effect can’t be reached in our study.

Therefore, I changed to use more conservative wording in the text, deleted some arbitrary sentences, and revised relevant reference of anti-cancer in HNSCC. I consider that making a balanced discussion between the effects of ARBs would be better than presenting only one side. I hope you could accept my explanation.

The followings are some changes made in our text:

1. We **deleted** the sentence” The effectiveness of anti-cancer therapies has been supported in many clinical studies.” **(Page 17 Discussion).**
2. We **deleted** the sentence” The results of our clinical study particularly highlight the anti-cancer effects on HNSCC;” **(Page 19 Discussion).**
3. We **deleted** the sentence” the ACEI perindopril has been shown to reduce the growth of head and neck squamous cell carcinoma (HNSCC) in vivo [26], suggesting a role for Ang II in HNSCC.” **(Page 19 Discussion).**
4. We **deleted** the sentences” Some might argue that the survival benefit from ARB use was probably from the anti-hypertensive effect instead of the anti-cancer effects. According to a previous study, patients with hypertension had poor long-term survival than those without hypertension [31]. However, in our study, 180-day ARB use in patients with hypertension was consistently associated with improved OS compared to patients without hypertension. (Crude HR_ARB_ users vs. non-HTN = 0.70, 95% CI = 0.50-0.99; adjusted HR_ARB_ users vs. non-HTN = 0.67, 95% CI = 0.48-0.95). The above findings displayed the possible anti-cancer effect of ARBs in oral cancer patients.” **(Page 20 Discussion).**
5. We balanced the discussion as followings: “…This was approved by Lin et al., which ARBs were found to exert antiproliferative and antiangiogenesis effects by inducing apoptosis in nasopharyngeal carcinoma (NPC). In addition, improved 5-year OS and DSS were found among patients with NPC using ARBs [27]. Interestingly, we found that ARB use for at least 180 days improved the OS rate of patients with OSCC statistically, yet not DSS rate (Table 5). Accordingly, RAS is a major regulator of blood pressure (BP) and vascular response to injury. There is large evidence that RAS inhibition provides end-organ protection independent of BP lowering [28]. That probably explained the survival benefit of using ARBs is mainly through as end-organ protective effect, which further reduced overall mortality [29].As for the anti-cancer effect of ARBs in oral cancer patients, it still remained controversial in our current study.” **(Page 19 Discussion).**

*Ref 1. George, A.J., W.G. Thomas, and R.D. Hannan, The renin-angiotensin system and cancer: old dog, new tricks. Nat Rev Cancer, 2010. 10(11): p. 745-59.*

*Ref 2. Hinsley, E.E., et al., Angiotensin 1-7 inhibits angiotensin II-stimulated head and neck cancer progression. Eur J Oral Sci, 2017. 125(4): p. 247-257.*

*Ref 3. Lin, Y.-T., et al., Angiotensin II receptor blockers valsartan and losartan improve survival rate clinically and suppress tumor growth via apoptosis related to PI3K/AKT signaling in nasopharyngeal carcinoma. Cancer, 2021. 127(10): p. 1606-1619.*
